# Can home visits for early child development be implemented with sufficient coverage and quality at scale? Evidence from the SPRING program in India and Pakistan

**DOI:** 10.3389/fnut.2023.1152548

**Published:** 2023-06-19

**Authors:** Zelee Hill, Shamsa Zafar, Seyi Soremekun, Siham Sikander, Bilal Iqbal Avan, Reetabrata Roy, Sarmad Aziz, Divya Kumar, Nazia Parveen, Shumaila Saleem, Deepali Verma, Kamal Kant Sharma, Jolene Skordis, Assad Hafeez, Atif Rahman, Betty Kirkwood, Gauri Divan

**Affiliations:** ^1^Institute for Global Health, University College London, London, United Kingdom; ^2^Fazaia Medical College, Air University, Islamabad, Pakistan; ^3^London School of Hygiene and Tropical Medicine, London, United Kingdom; ^4^Global Institute of Human Development, Shifa Tameer-e-Millat University, Islamabad, Pakistan; ^5^Sangath, Goa, India; ^6^Health Services Academy, Islamabad, Pakistan; ^7^Institute of Population Health, University of Liverpool, Liverpool, United Kingdom

**Keywords:** early child development, home visits, implementation, process evaluation, community health workers, community based agents, India, Pakistan

## Abstract

**Introduction:**

There is limited evidence from low and middle-income settings on the effectiveness of early child development interventions at scale. To bridge this knowledge-gap we implemented the SPRING home visiting program where we tested integrating home visits into an existing government program (Pakistan) and employing a new cadre of intervention workers (India). We report the findings of the process evaluation which aimed to understand implementation.

**Methods and materials:**

We collected qualitative data on acceptability and barriers and facilitators for change through 24 in-depth interviews with mothers; eight focus group discussions with mothers, 12 with grandmothers, and 12 with fathers; and 12 focus group discussions and five in-depth interviews with the community-based agents and their supervisors.

**Results:**

Implementation was sub-optimal in both settings. In Pakistan issues were low field-supervision coverage and poor visit quality related to issues scheduling supervision, a lack of skill development, high workloads and competing priorities. In India, issues were low visit coverage - in part due to employing new workers and an empowerment approach to visit scheduling. Coaching caregivers to improve their skills was sub-optimal in both sites, and is likely to have contributed to caregiver perceptions that the intervention content was not new and was focused on play activities rather than interaction and responsivity - which was a focus of the coaching. In both sites caregiver time pressures was a key reason for low uptake among families who received visits.

**Discussion:**

Programs need feasible strategies to maximize quality, coverage and supervision including identifying and managing problems through monitoring and feedback loops. Where existing community-based agents are overstretched and system strengthening is unlikely, alternative implementation strategies should be considered such as group delivery. Core intervention ingredients such as coaching should be prioritized and supported during training and implementation. Given that time and resource constraints were a key barrier for families a greater focus on communication, responsivity and interaction during daily activities could have improved feasibility.

## Introduction

The importance of early child development (ECD) interventions to improve short and long term outcomes and disrupt the intergenerational cycle of poverty is well recognized (1–5). ECD holds a central place in the Sustainable Development Goals, with multilateral support for implementation and scale up (6–9). Global guidelines recommend that all infants and children should receive responsive care and have early learning activities with their caregivers in the first 3 years of life (7). Interventions to achieve this include providing support for caregivers through individualized home visits and/or holding group sessions at home or in community spaces or through contacts at health facilities (5). In low and middle income settings interventions are most commonly community based and delivered by peer educators or community based agents (CBAs) who have some high school education and 1 to 2 weeks of ECD training (10).

We know from efficacy trials that community ECD interventions can improve child development outcomes in diverse settings, and can offset developmental risks such as poverty and malnutrition (5, 11, 12). However, evidence is lacking on effectiveness at scale, with a major gap in research on the ‘how to’ of ECD interventions (13–17). This lack of implementation evidence has been cited as a reason for the slow roll out of ECD programs in low and middle income countries (18). To support scale up we need to share implementation successes and failures and understand what processes and factors contribute to positive impacts, or lack of impact, in relation to the workforce, infrastructure needs, quality, coverage and demand (13, 16, 17, 19, 20).

To bridge the scalability knowledge-gap we implemented the SPRING program, which was designed from the outset to be feasible, affordable and appropriate for delivery at scale. SPRING designed and tested the impact of monthly home visits by CBAs to promote and support caregivers with responsive care, early learning and nutrition practices in India and Pakistan using a cluster randomized controlled trial design (21, 22). SPRING adapted WHO and UNICEF ECD and nutrition content (23, 24), and tested two delivery models for scale-up using CBAs: In Pakistan the intervention was called Roshan Kal (brighter tomorrow) and was integrated within the government Lady Health Workers program (25). In India the intervention was called Kilkaari (gurgling of a young child) and was delivered by a non-governmental organization (SANGATH) who employed a new cadre of intervention specific CBAs called Kilkaari workers (KWs). We refer to the Roshan Kal and Kilkaari programs collectively as the SPRING Program.

As reported in our companion paper, the SPRING Program did not achieve significant impacts on ECD or growth outcomes at 18 months of age (22). It is important to understand why this occurred, so that others can learn lessons for delivering community based ECD and nutrition interventions at scale. Embedded in the SPRING program was a process evaluation that aimed to understand implementation in relation to training, supervision, coverage, quality, acceptability and barriers and facilitators to behavior change among CBAs and families. In this paper we focus on the process evaluation findings that relate to ECD as this is where evidence is most lacking.

## Materials and methods

### The intervention

In Pakistan, SPRING was embedded in the government Lady Health Worker (LHW) program. The LHW program was established in 1994 and LHWs are well known and credible health workers in their communities. The LHW role comprises over 20 maternal and child health services including health education on breastfeeding and complimentary feeding, child growth monitoring, immunizations, family planning and basic curative care. Within the government program LHWs receive 15 months training, are aged 18–45 years, resident in the community they serve, have a minimum 8 years of education, are preferably married and are acceptable to the community. Each Union Council has 15–20 LHWs, each serving 150–200 households, they are supervised and monitored by a Lady Health Supervisor (25). LHWs conduct approximately 7 home visits per day and the SPRING content was integrated into these home visits.

In India the KWs were lay workers specifically identified, recruited, trained and supervised to only deliver the SPRING intervention. KWs were recruited by SANGATH with renumeration and characteristics similar to government frontline workers. They were resident in the community, had a minimum of 8th grade education, were married and had good communication skills. Each served a maximum of 100 eligible households. As new workers they had to identify eligible families so were asked to engage with local frontline workers and attend local community events such as Village Health Days to identify new pregnancies and mothers with young children.

In Pakistan SPRING was implemented in 10 predominantly rural Union Councils in Rawalpindi District by 150 LHWs; 70 of these LHWs and the children in their catchment areas were included in the SPRING outcome evaluation. In India, SPRING was implemented in three blocks of the predominantly rural Rewari district in Haryana State by 54 KWs. The intervention was delivered to all pregnant women and families with children 0–2 year of age. The content (Table 1) was adapted based on formative research into current behaviors, and barriers and facilitators for adopting the desired ECD and feeding practices (26). In pregnancy, home visits focused on maternal health and sensitization about breastfeeding. Postnatal visits focused on caregiver-child interaction and play activities, breastfeeding, complementary and responsive feeding, with new messages introduced each month dependent on the age if the child.

**Table 1:** Key SPRING content.

**Age at visit**	**Nutrition related**	**Interaction and play (being responsive, follow child’s lead, focusing on child, scaffolding, praise)**
**Pregnancy**	Importance of/encouraging family support and involvement	
	Iron, diet and rest during pregnancy	Importance of play and interaction
	Early and responsive breastfeeding with love and care	
	No pre-lacteal feeds	
**Neonatal period**	Exclusive and responsive breastfeeding with love and care	Talking/singing and looking into the child eyes while breastfeeding or doing daily activities, caressing and cuddling, allowing freedom of movement of limbs
	Avoiding insufficient milk	
**1-5 months**	Exclusive breastfeeding until 6 months and then continued breastfeeding	Interacting through activities such as: following objects, grabbing and exploring objects, making sounds with rattles and by banging objects, singing, copying sounds and movements
	In the last visit before 6 months, introduce concept of weaning from 6 months	
**6-11 months**	Continued breastfeeding	Interacting through activities such as: peek a boo, rolling a ball, physical play such as bouncing, naming body parts and objects, finding objects, imitating actions
	Age dependent complementary feeding messages on: Responsive feeding Frequency, variety, consistency, quantity Hygiene Adding “super” foods (e.g. butter or oil) Finger food/self feeding Enjoyable meal times Avoiding “junk” foods (Pakistan only)	
	Importance of feeding for a sick child	
**Second year**	Continued breastfeeding	Interacting through activities such as: putting in and out, stacking, singing, following instructions, color matching, mark making, pulling, sorting, counting
	Eating with the family and balanced diet	

SPRING was delivered using a counseling approach based on Cognitive Behavior Therapy techniques (27) which includes: family support; guided discovery using pictures; behavioral activation; empathic listening; problem-solving; and praise. Structured pre-tested counseling cards provided pictures for families to look at and instructions and key messages for the CBAs. In each visit approximately 4 messages were given – e.g. in postnatal visits this was two on nutrition and two on play and interaction with the infant. In Pakistan routine program messages were integrated into these counseling cards and families were also provided with a reminder calendar. The visits targeted the primary caregiver, and included providing information, problem solving and family engagement. The primary caregiver practiced play activities and received coaching and demonstrations from the CBA to enhance skills, self-efficacy and recall in relation to play activities and responsivity. Community and health systems sensitization was also conducted.

CBAs received 8 days of skills based training, delivered in two phases to groups of 14–29 CBAs by SPRING supervisors – female social science graduates trained by a child development expert. Training included classroom sessions, participatory exercises, role play, discussions of videos and practical sessions with caregivers and children.

Monthly group supportive supervisory meetings were held in groups of 11–25 CBAs who shared experiences and supported each other, problem-solved and developed their skills. In Pakistan this was integrated into the LHW’s routine monthly supervision meetings. During monthly one-to-one supportive field supervision, supervisors observed home visits and used a supervisory checklist to feedback on performance, problem solve and make suggestions on areas for improvement. The CBA to supervisor ratio was around 1:10 in India and 1:15 in Pakistan.

### Process evaluation data collection

#### Quantitative data

Details of the quantitative process data collection can be found in Table 2. In brief, training knowledge gain was determined using a pre- and post-training test, group supervision coverage from program attendance record and field supervision from completed supervisory checklists. Data on visit coverage data came from the SPRING surveillance system (22), implemented as part of the outcome evaluation, under which resident surveillance workers visited households every 8–10 weeks to collect outcome data. Data on visit quality were obtained from a supervisory checklist of 33–37 items, dependent on site and whether a pregnancy or child visit was being observed. The checklist covered counseling, family support, problem-solving and child stimulation with between 3 and 15 items for each of these domains. The checklist questions were not identical in each site, and to aid comparison we present a selection of overlapping indicators for each domain. Information on visit duration and the use of job aids was collected in a cross-sectional survey with caregivers.

**Table 2:** Quantitative process data collection methods.

Process domain	Data source	Content	Sample (n) = number of respondents
Training knowledge	Self -administered pre- and post-training multiple choice test	Knowledge of counseling, nutrition, play and interaction	All KWs (40) and LHWs (149) who attended the 8 days SPRING training
Group supervision coverage	Program attendance records	Number of supervision groups held and percent CBAs that attended	All KWs active during the evaluation period (n = 62) and evaluation zone LHWs* (69)
Field supervision coverage	Supervisor checklists completed	Percentage CBAs with a completed checklist	All KWs active during the evaluation period (n = 62) and evaluation zone LHWs* (69)
Visit coverage	Surveillance data from all women who had a pregnancy or infant under 2 years of age during the evaluation period	India: Visit by a maternal and child health worker in the last month and who they were** Pakistan: Visit by an LHW in the last month	India: 3446 women and 29,860 questionnaires Pakistan: 3237 women and 24,772 interviews
CBA skills	Supervisory checklists used in monthly observed supervision visits during the evaluation period	Percentage CBAs with a completed checklist with skills in: Counseling, family support, problem-solving and child stimulation.	All KWs active during the evaluation period (62 KWs and 1,398 checklists) and evaluation zone LHWs with at least one checklist completed* (68 LHWs with 381 checklists)
Duration of visit and use of job aids	Endline survey with primary caregiver	Duration of last visit and use of job aids	India: All 661 women with children under 2 years of age Pakistan: 120 randomly sampled women with children 0–11 months of age.

*Supervision focused on the evaluation zones in the last year of the program.

** Asked this way as the KWs in India were new and less easily identified.

#### Qualitative data

Data on acceptability of the intervention for families and the CBAs, and barriers to and facilitators for change were collected toward the end of implementation from four intervention clusters in each country. Data were collected by trained bilingual interviewers, who were not part of the implementation team. The clusters were selected to ensure a range of home visit coverage, and to reflect the diversity of the study area. Data were collected through narrative in-depth interviews (IDIs) with mothers and focus group discussions (FGDs) with mothers, grandmothers, and fathers. Supervisors were interviewed through FGDs in Pakistan and IDIs in India. Sample size was approximated based on when saturation had been reached in our previous studies in the area, and additional interviews and FGDs were added if needed. The methods, sample size and content are shown in Table 3.

**Table 3:** Qualitative data collection methods, sample size and content.

Method	Sample size per site	Content covered
Mother narrative in-depth interviews	14 in India 20 in Pakistan Divided between infants 7–9 months and 16–20 months	Description of behaviors and the contexts within which they occur Contacts with CBAs, and attitudes to visits and visit content
Mothers FGDs	4 FGDs	Acceptability and response to the intervention and to CBAs Barriers and facilitators to behavior change
Father and grandmothers FGDs	2 with each group in India 4 with each group in Pakistan	Acceptability and response to the intervention Role in intervention visits Role in play and feeding behaviors Barriers and facilitators to behavior change
CBA FGD	4 FGDs 2 additional FGDs and 2 IDIs in India to explore coverage	Issues affecting coverage and quality of visits Successes and challenges of the intervention Impact of intervention on their life and other work Barriers and facilitators to behavior change
Supervisor IDIs/FGDs	3 IDIs in India 2FGDs in Pakistan	As for CBA
Stakeholder IDIs	5 in Pakistan	Acceptability of the visits Perceived impact of the intervention on LHWs work Views on integration between SPRING and other activities

IDI mothers were selected from the surveillance database to ensure that the sample had a variety of socio-demographic characteristics (age, education, parity, gender of the child, and socioeconomic status). Community FGDs participants were selected through community informants to have a range of parities, education levels and socio-economic status and stratified for homogeneity where mixed groups could inhibit participant interaction. Topic guides were developed in local languages and pilot tested before data collection. All Interviews and FGDs were audio recorded in addition to notes taken. Narratives and IDIs were turned into expanded notes using the audio recordings to add verbatim quotes (28). Debriefs were held regularly during data collection to provide feedback to interviewers and discuss themes and saturation. These discussions and the topic guides were used to develop a deductive coding template, with inductive codes and themes added during analysis. A framework analysis approach was used for the narrative IDIs, and thematic analysis using a phenomenological approach for the other IDIs and FGDs (29, 30). In the results when reporting on the individual countries we refer to LHWs and KWs and when reporting combined results we refer to CBAs.

## Results

### Training and supervision

Training increased overall ECD and nutrition knowledge scores among LHWs from 50 to 91%, and among KWs from 70 to 85%. Areas where knowledge was suboptimal post-training were targeted during individual and group supervision sessions. Group supervision coverage was high in both sites, with more than 75% of the monthly groups occurring and high attendance (see Table 4). In India mean monthly individual field supervision coverage was 91%, but only 23% in Pakistan, where each LHW had field supervision an average of 5.5 times (range 2–7 times) over the 24 months of implementation. Field supervision was made difficult in Pakistan as the number of days LHWs were available for supervision was limited due to polio activities, official meetings, training, maternal and child health week activities, measles campaigns, strikes and public holidays; with a new supervision round initiated only when all LHWs had been seen.

**Table 4:** Training, supervision and visit coverage.

	**India**	**Pakistan**
Pre and post training knowledge scores	70–85% (*n* = 46)	50–91% (n = 149)
% of monthly supervisory groups that occurred	100% (*n* = 24 months)	75% (*n* = 24 months)
Mean monthly supervisory group attendance (of groups held)	100% (*n* = 62)^*^	89% (*n* = 69)^**^
Mean monthly supervision coverage	91% (*n* = 62)	23% (n = 69)
% caregivers ever visited by a CBA	95% (*n* = 3,446)	100% (*n* = 3,237)
% of caregivers who received a CBA visit in the last month	30% (*n* = 3,446 women and 29,860 visits)	92% (*n* = 3,237 women and 24,772 visits)

*n refers to all KWs ever trained including replacements.

**n = LHWs in evaluation zones.

In both sites the CBAs valued SPRING supervision because it focused on feedback rather than on *“checking,”* allowed for collective problem solving and enhanced relationships with each other and their status in the community:

“In Roshan Kal [SPRING] supervision, LHWs are like a family and there is no hesitation, where as in the Lady Health Supervisor meetings they feel fear from ways of checking” [LHW FGD Pakistan]

“[The] Supervisor’s way of giving feedback is good. It does not hurt us. In fact we like it…. in group meeting supervisor asks everyone...so we get the solution from each other” [KW FGD India]

“Actually feedback was appropriate…they [KWs] never defended…..they have taken the feedback in a positive way” [Supervisor IDI India]

### Coverage of visits

Based on data from the caregiver surveillance system, most caregivers were visited by a CBA at least once (>95%) (see Table 4), indicating that CBAs were able to locate families. In Pakistan the mean monthly visit coverage was 92% (monthly range 78–98%), but this was for any LHW visits including, for example, polio visits. In India only 30% of caregivers reported a visit in the last month (monthly range 20–45%), with coverage similar by age of the child and by socio-economic status but varied by KW cluster (ranging from 12 to 50%).

Supervisors in India identified 43% of KWs as either generally poor performers in relation to coverage or of facing issues such as a high load of migrant population, being replacement workers, lack of family support, taking sick leave and long travel distances. The KWs themselves did not perceive coverage as an issue, but when asked about reasons for low coverage they reported isolated households, the lack of safety in certain neighborhoods and a large workload reducing their ability to make frequent visits. Some households refused visits either because a household elder did not want the visit to occur, the mother themselves did not want it or because visits were not seen as needed. It was harder to find mothers during harvest time:

*“Respondent 8*: Maybe her [KWs] family refuses her or she has less time

*Respondent 6*: She doesn’t get family support or her mother in law is upset

*Respondent 3*: Maybe she is busy in other work

*Respondent 8*: Maybe she has young children…. She isn’t able to manage the time” [KW FGD India]

“The mother does not allow outsiders [to] visit her house. She doesn’t go anywhere- always stays at home” [KW FGD India]

“During harvesting, mother goes in field – some don’t go but many of them had to go… fewer visits happen” [KW FGD India]

KWs reported that they enjoyed conducting visits, particularly because of the identity and status that the role gave them: *“We have got a good identity in the houses. Earlier we did not used to go out, no one knew us. Now everyone knows”* [KW FGD India]. For some, it was their first paid employment outside the home and they described the role as transformative in terms of increasing their knowledge of the outside world and their ability to navigate it: *“I never went to the village alone but after I joined here…. I do not hesitate to go anywhere in the village”* [KW FGD India]. The financial independence the role gave them was also key for some workers: *“My husband drinks a lot. With the salary I pay children’s school fee and manage household expenses. …Earlier I used to cry and stayed at home….. Now I am managing everything by my own”* [KW FGD India].

### Quality of visits

In India, home visits were SPRING specific, so if a home visit occurred we can assume that SPRING content was covered. In Pakistan SPRING content was added to existing visits and the high visit coverage may not reflect high SPRING content coverage. In the Pakistan endline survey (n = 120) 36% of mothers reported that their last visit was under 10 min long and only 29% that counseling cards were used, suggesting that coverage of SPRING content was most likely suboptimal. The SPRING protocol was more closely followed in India with women reporting longer visit lengths and 78% reporting that counseling cards were used in every visit.

Table 5 shows data from the field supervision observation checklist. Observed counseling skills were high in both settings, with the exception in Pakistan of using positive words and gestures, which was only observed in 53% of the supervision visits. In Pakistan the LHWs reported in the qualitative interviews an improvement in their overall approach to counseling: *“In the past, we just asked about a behavior, like whether mother has breastfed or not, now we explain in detail and tell the advantages of it and discuss the difficulties as well”* [LHW FGD Pakistan]. For the KWs their new counseling skills were also valued with impacts on their self-confidence: “*We have learnt a way of talking, developed self-confidence among people. So, I really liked to work here*” [KW FGD India].

**Table 5:** Skills observed during supervision.

	India (62 KWs and 1,398 checklists)	Pakistan (68 LHWs and 381 checklists)
*Counseling*		
Greets the mother/family	94%	99%
Seat herself appropriately	93%	94%
Makes eye contact and nods during discussions	91%	95%
Uses positive words and gestures when mother says something right	83%	53%
*Use of counseling cards*		
Shows at least two pictures and asks the mothers opinions on them	98%	87%
Uses probes allowing mother to describe the picture/asks mother about the pictures	75%	70%
*Visit fluency*		
Fluent with the content	88%	48%
Covers all relevant topics	-	28%
*Family engagement/support*		
Welcomes family members to sit in and be part of the session	84%	51%
Encourages key family members to support the mother and infant:	65%	54%
*Problem solving*		
Asks about problems from previous visits	85%	68%
Asks about potential problems for current messages	67%	24%
*ECD and coaching* (for children >2 months old)		
Asks mum to demonstrate/discuss last visit’s activities/Asks about activities the child has been doing since the last visit	75%	75%
Selects appropriate new activity	96%	74%
Clarifies/discusses benefits of the activity	86%	38%
Observes caregiver trying the activity	-	82%
Guides mother in trying the activity	–	40%
To be responsive	52%	–
To be focused	58%	–
To follow the child’s lead	44%	–
To praise the child	73%	–
Prompts caregiver to encourage child	63%	50%
Prompts caregiver to praise the child’s efforts	73%	36%
Models a behavior (e.g. clapping when child does something well)	59%	31%

The use of counseling cards during observed supervision visits was high in both settings, but visit fluency was much lower in Pakistan than India (48% observed as fluent compared with 88% in India), and in Pakistan all relevant topics were covered in only 28% of observations. Family engagement and problem solving were also lower in Pakistan than India as were ECD and coaching skills, with guiding, prompting and modelling particularly low in Pakistan (31–52%). In India there were striking improvements in ECD related skills over time. For example, guiding the mother to be responsive increased over the course of the intervention from 24% in the first supervision round to 75% in the final round, guiding to follow the child’s lead increased from 19 to 47% and modelling behaviors from 35 to 79%. In Pakistan only observing the caregiver trying the activity (increased from 85 to 91%), prompting the caregiver to encourage the child (40–66%) and modelling behaviors (20–49%) increased over time, while skills related to family involvement, showing counseling cards and problem solving decreased over time in Pakistan.

Mirroring the quantitative results, caregivers in the qualitative interviews reported better use of counseling cards and the provision of messages in India compared to Pakistan. For example, in India most caregivers reported that counseling cards were used in the visits, that they were asked to describe the pictures which were then explained by the KW and they recognized most of the counseling cards. Caregivers less frequently reported that they were asked to try play activities with the KW present. In Pakistan caregivers reported that counseling cards were rarely used and that the play component was often limited to general advice to play more or a verbal description of an activity to try:

“LHW didn’t tell any proper play activity .... told them [family] to give time to child” [Mother FGD Pakistan]

“LHW told us to play with the child to make her intelligent.... LHW also advised us to give blocks to the children.” [Mother IDI Pakistan]

Both the LHWs and the supervisors corroborated the mothers’ descriptions of visits, reporting that the use of counseling cards and coaching/demonstrations were rarely done. There were some exceptions where mothers described being told about new activities with counseling cards and/or being guided to try them:

“I gave cups and glasses to my son and taught him to recognize things. This was new for me and I didn’t know about it when I had my older child. LHW explained these activities to me through pictures” [Mother narrative Pakistan]

### Barriers to conducting high quality visits

In the qualitative interviews, CBAs in both settings recognized the importance of appropriate counseling, family engagement, praise, and coaching and could describe high quality visits, reflecting that they knew what they should be doing: *“LHW should pay greetings, introduction of Roshan Kal* [SPRING]*, family involvement, health calendar follow up, follow up of the play activity, which benefits they got from these messages, show pictures, ask about the pictures, tell about the picture, asking about problems, keep involving family and to conduct the play activity”* [LHW FGD Pakistan].

The CBAs felt that new skills, especially ECD skills, took time to develop: “*We like the child play activities, but we only came to know about these through Roshan Kal [SPRING] trainings, this is something new and we will take some time to do these perfectly*” [LHW FGD Pakistan]. The lower quality visits in Pakistan were mainly attributed to a lack of LHW time due to competing activities such as polio and dengue, delivery referrals, mother and child weeks and trainings and meetings: “*We want to, but we cannot deliver Roshan Kal [SPRING] visits as planned because of program activities*” [LHW FGD Pakistan]. SPRING content was less of a priority than activities that had a direct impact on their salaries or were monitored by the program: “*We have to bring delivery referrals to the health unit, which takes a lot of our time. If we do not do that our salaries are deducted*” [LHW FGD Pakistan]. There were also issues around the LHWs assuming they did not need to focus on play in educated families or where families reported they already played with their children, caregivers being busy or uninterested, children being asleep, absent or not in the right mood and LHWs being embarrassed to coach:

“LHWs don’t conduct play activities because of workload and because they think verbal messages are enough” [LHS FGD Pakistan]

“If mother says that she is doing, they believe in her and end the visit” [LHW FGD Pakistan]

*“Respondent 1*: People make fun of her, mothers says that they are already doing all this

*Respondent 2*: People start laughing when LHW tell them about play*....*

*Respondent 3*: People say what is this new thing?” [LHW FGD Pakistan]

In India, the KWs were SPRING specific and so had no competing activities. The main quality issues were related to inconsistently demonstrating, modelling and promoting ECD behaviors, mainly attributed to caregivers not having time or the KW perceiving them as being busy and to the KWs being poorly received:

“We [KWs] give enough time to the mother, but mother is in hurry” [KW FGD India]

“If mother was doing some work …and left it - then mother-in-law gets angry for not completing the chore first. Sometimes they get angry in front of us” [KW FGD India]

“Her husband saw us and went outside. Next time he came in…. he started grumbling. The husband got angry that she [mother] hadn’t cooked the chapatti yet.” [KW FGD India]

When mothers describe the KW visits, they more frequently reported that the KW asked them to show them the activity they had been doing previously rather than trying and being coached on the new activity, this was seen as “*checking*” what they had done. This “*checking*” was not included in the KW counseling cards, which focuses on trying and coaching around the new activity, but it was on the supervisor checklist.

### Acceptability of visits

Overall, the CBAs were accepted and trusted by most families they visited. In both sites they were described as “*patient,” “helpful,”* and *“supportive”* and gave *‘good’ and ‘right’* information *‘nicely’, ‘calmly’* and *‘jovially’.* Most mothers described the visits as being pleasurable and valued that someone else was concerned about their child: “*There was another person to care about upbringing the child. We look after the child but when she comes, I feel different – it feels good”* [Mother FGD India].

In Pakistan the LHWs were long standing workers who were well know and trusted in their communities. In India the KWs were newly recruited and trust and recognition took time to develop: *“Initially when we used to go for a visit, mother did not pay attention…they used to ignore* [what we said]. *But now… they listen to us carefully and also give respect saying sit for a while, have tea…”* [KW FGD India]. By the end of the intervention acceptance was high, but both community respondents and the KWs reported that it was not universal with some families finding their KW too young and inexperienced and that visits imposed on their time.

Families liked the counseling cards and, in Pakistan, the calendar. Families found them salient and felt they enhanced understanding*: “We had some knowledge and some we gained through pictures… It was easy to understand through pictures”* [Mother FGD India]…. *“When I looked at it, I said well this is my situation”* [Mother IDI Pakistan]. The benefit of using counseling cards was echoed by the KWs, especially for illiterate women: *“Without picture they will not have understood so much. They understood more through pictures”* [KW FGD India].

Grandmothers often looked after the child and SPRING aimed to encourage fathers and other family members to support the mother and interact with the child, but as seen in the supervisor checklist data family engagement in the visits was low in both sites. Family members were not always at home, but the qualitative data also showed that some grandmothers and fathers were reluctant to be involved in the visits due to time constraints or because they did not feel the visits were important for them, and for fathers in India because it would mean the KW would have to wear a veil during the visit:

“Sometimes it is only the mother-in-law who plays with the child….. Mother ….. says, tell mother-in-law, let mother-in-law try the activity …. mother-in-law says, tell her [mother] only. I am uneducated. I don’t know” [KW FGD India]

"Has to veil her face from male members of the house … that’s why male members also don’t join" [Mother narrative India]

In Pakistan LHWs said another reason they did not involve families was because this added time to their visits and because not all of the content of their routine visits was appropriate for other family members: *“They could not involve other family members because of workload …. they just told messages to mothers mostly and do not involve family members”* [LHW FGD Pakistan].

### Barriers and facilitators to family uptake

In both sites most caregivers reported that the visits had increased the amount of attention they gave their child and their knowledge of the importance of play, but families rarely reported that they had done the play activities routinely and frequently. Most reported that they did each activity once or twice or for a few days.

Among those who did the activities regularly the main drivers were trusting the CBAs, knowledge of the benefits of play and positive experiences/outcomes such as enjoyment by child and mother and pride that a child could to the activity:

“We thought if KW is saying then we can try..... they are saying for some benefit” [Mother narrative India]

“The child will be more intelligent and will pick quickly when she will join school” [Mother narrative Pakistan]

“She plays 2-3 times a day with child and the child enjoys a lot and she can also feel the child’s happiness from his face” [Mother IDI Pakistan]

“When I make her learn and she does, then I feel like doing more things with her. I feel very happy” [Mother narrative India]

Barriers to family uptake are shown in Table 6, with illustrative quotes. In both sites all respondent groups reported caregiver time pressures as a key reason ECD activities were not done. Caregivers were often in charge of most domestic chores, including cooking, heating up water for baths, hand washing laundry, care of the domestic livestock and harvesting twice a year. Time pressures lead to other household members looking after the child who, as described above, were infrequently engaged in the visits: *“After doing all chores, I think to make child sleep and take rest. Mother-in-law and father-in-law are there. They play a lot with the child”* [Mother FGD India].

**Table 6:** Barrier to families conducting ECD activities.

*Lack of time:*
“Each day we have some or the other work and get little time for children” [Mother narrative India]
“Women have more work to do and no free time to follow advice about play activities..... and then children can start playing by themselves” [Mother FGD Pakistan]
*Perceived lack of difference or novelty:*
“There is no effect, we play the same way as with elder children …. we knew it earlier …... At that time we never cared much about doing them” [Mother narrative India]
“Mother says that ‘don’t we know that how to play with our children? We are doing this generation after generation’” [LHW FGD Pakistan]
*Limited salience of long-term impacts*
“[The benefit is] so that he identifies the things and understands that he can play with them” [Mother IDI India]
“When child will play she will not cry and if she does not cry she will be healthy… The child is too young to learn something from the play” [Mother narrative Pakistan].
*Child will learn in their own time:*
“The child learns everything without any guidance....will learn by looking at you” [Mother narrative India]
“Leave it! Later when child will grow, he will be able to understand the activities and do them by own” [Mother narrative India]
*Child is too young for the activity:*
“Tried this activity 2-3 times but the child was unable to put things inside” [Mother narrative India]
“LHW tells me to give my child blocks to play and tell her about colours through them, but I feel my child is too young for it” [Mother IDI Pakistan IDI-mother]
*Child learnt the activity:*
“Why to repeat same thing with the child [laughs]….If there is something new, we can do …. Child doesn’t do same activity again [laughs]… she learnt” [Mother narrative India]

Few families reported the potential for play and interaction to have long term effects, instead focusing on the child learning a new ability and the activity keeping them happy or distracted. Few felt that the CBAs was suggesting a new behavior, with play something that was already done, and caregivers focused their descriptions of the visits on the play and play activities being promoted rather than the responsivity and interaction that doing the activity was aiming to enhance. Other barriers were beliefs that the child would learn in their own time without interactive play, that families desired children to play independently so they can occupy themselves, and a perception among some caregivers that their child was too young for the suggested activity, or conversely that their child could do the activity easily.

**Figure 1:**
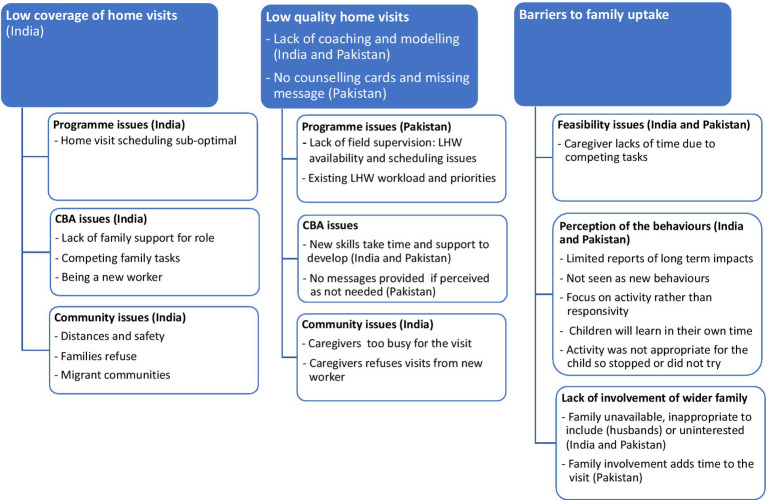
Summary of the barriers to coverage, quality, and uptake.

The key barriers to coverage quality and family uptake are summarized in Figure 1.

## Discussion

The SPRING intervention was based on existing global guidelines, was designed using best practice in relation to formative research and counseling practices and included elements considered key to the success of ECD interventions such as a structured curriculum, actionable messages, opportunities for caregivers to practice activities and get feedback and coaching, demonstration, problem solving, skills based training and supportive supervision (10, 23, 24, 26, 27). SPRING was designed from the outset to be feasible, affordable and appropriate for delivery at scale, and thus to answer key questions about effectiveness at scale and implementation processes. Using a rigorous cluster RCT design we found no impact on ECD or growth outcomes neither when SPRING was integrated into the Lady Health Worker program in Pakistan nor when it was delivered by intervention specific NGO workers in India (22). It is essential to identify what could have caused this lack of impact given the consistent impact seen in efficacy trials (5) and the call for ECD interventions to be scaled up (7).

Key implementation issues were low supervision coverage and poor fidelity in Pakistan, and low visit coverage in India. In addition, the coaching element of the intervention was sub-optimal in both sites, which is likely to have contributed to caregivers feeling that the intervention was not promoting anything new and that visits focused on play and play activities rather than on the interaction and responsivity - which was to be the focus of the coaching. This is important as interventions that promote responsivity have a greater impact than those that focus only on play and learning materials (5).

Supervision of community health workers is often weak and under-supported with low and irregular coverage and an administrative focus (31). We were able to implement a supportive group supervision system in both settings which was well received by the CBAs. Individual field supervision focused on improving the quality of the visits through feedback and problem solving, but coverage was very low in Pakistan, which may in part explain the low content fidelity. In India individual supervision coverage was high and we saw ECD skills starting low and improving over time, this was seen to a lesser extent in Pakistan. Other studies have also found that skills take time to develop as workers need to become familiar with the new approach and be supported through supervision in order to complete all the objectives and cover key concepts in their visits (13, 32). The low supervision coverage in Pakistan was in part due to the realities of the LHW schedules and commitments, which needed to be taken into account more fully when designing the supervision system. Other home visit ECD interventions in Pakistan using LHWs have had good supervision coverage, so with the right system high coverage is achievable (13). SPRING field supervision in Pakistan required a better balance between ensuring that all LHWs were seen in a supervision round (leading to potentially long gaps between rounds), this could have been achieved through conducting monthly supervision for all available LHWs, but with a special focus on those missed the previous month.

In many settings it may be impossible for existing CBAs to cover the population in need (33–35) and new workers may need to be deployed. Utilizing an NGO model, with project specific CBAs who have no competing work tasks and carefully delineated catchment areas, does not guarantee adequate coverage with only 30% of eligible women in India reporting a home visit in the last month. The low KW visit coverage in India was in contrast to the 96% coverage achieved by the SPRING Surveillance workers in the same sites. KW and Surveillance workers were similar in their education, pay, catchment area and access to transport. Their management structures differed with the Surveillance workers having independent checks on their coverage and performance and a more structured approach to scheduling their visits with mothers. The KWs, following an empowerment model, had more flexibility in scheduling their visits and fewer independent checks. A program review of 10 country experiences of scaling up CBA postnatal home visits demonstrate the difficulty of achieving high coverage at scale, with most countries achieving less than 10% coverage and none achieving more than 20% coverage (36); the magnitude of this challenge should not be overlooked. Programs need strategies to maximize coverage, including having a strategy for scheduling visits and identifying and managing poor performers through monitoring and feedback loops for course correction.

The experience of scaling up Crianca Feliz in Brazil, which found no impact on a range of ECD outcomes (37), was that rapid scale up was a barrier to achieving quality and consistency; this is an important example that coverage should not be prioritized at the expense of quality (38). In Pakistan LHW coverage was impressively high, but visit quality was low. As described above this was likely in part due to poor field supervision, but interacting with this were issues of integrating additional ECD tasks into the LHW workload. Resonating with our findings other studies have found that workforce constraints such as limited staff, high workloads, competing tasks seen to be of higher priority (e.g., immunizations), incentives for other work, large catchment areas and lack of supervision and remuneration structures inhibited coverage and quality visit by CBAs (32–35). There are clear synergies of integration in relation to efficiencies, having a worker who can provide holistic care and understands the needs of the whole child and family (20), but where existing CBAs have low coverage, are overstretched and systems strengthening is unlikely then alternative implementation strategies included in the WHO global guidelines should be considered such as group delivery, primary health care contacts or a mixture of these (7).

The LHW program was going through a difficult period during SPRING implementation which was likely to have influenced LHW motivation and ability to take on new tasks. This included fallout from the abolition of the Federal Ministry of Health and the devolution of activities to provinces in 2011, and regularization of LHWs in 2012 (39–42). The SPRING study team recorded resulting changes in the program that affected implementation which included: an increase in the catchment population of LHWs from 1,500 to 2000 after regularization, 24-h delivery services at Basic Health Units leading to increased workload for LHW as they were to accompany women to the facility, difficulties for SPRING staff engaging with the provincial government, and delays in the disbursement of salaries to LHWs.

In both sites the qualitative data suggest that coaching and modelling of behaviors was low, and that caregivers saw the intervention to be related to play and play activities rather than to responsivity and interaction. Core intervention ingredients need to be made clear to programs for prioritization during training and implementation and steps put in place to fully support these, especially where they are time consuming and may be the first thing to be dropped by busy workers. For example, the practical coaching of families in responsive care has been identified as a critical component of successful interventions, but this task takes time and skills and may be new to CBAs. We saw that in India, with frequent field supervision, KWs ECD skills increased over time and that acceptability also took time to develop–KW workers would have benefitted from a longer embedding period before the child outcomes were evaluated.

Mothers in the qualitative study reported a change in the attention they gave their children and as has been found in other studies most mothers liked the visits and enjoyed the play activities (13, 32, 34, 38). But, they rarely reported doing the play activities routinely and frequently. Time and resource constraints were a key barrier and although several activities were based around everyday activities a greater focus on communication, responsivity and interaction during daily activities could have improved feasibility. Few caregivers reported any perceived long-term benefit of play or saw the intervention as promoting anything new – perhaps related to the lack of coaching and modelling – and thus they had inadequate motivation to engage in the activities given their busy lives. Future programs need to ensure that interventions include a strong motivational element, ensure interaction and responsivity come across strongly and clearly communicate how the behaviors being promoted are different from existing behaviors. Utilizing approaches such as Trials of Improved practices (43), could provide tangible evidence of the impact of barriers such as time and resources and how these could be overcome.

Although the interventions were based on formative research, we adopted a relatively linear approach to implementation. Given the complexities of the contexts a pilot and having on-going adaptation, improvement and responsive feedback built into implementation would have given us the opportunity to refine the intervention and maximize impacts and should be built into future programs (44, 45).

The study had several strengths in that we could utilize coverage data from a rigorous surveillance system and use this to select a wide range of respondents for the qualitative component. The results of the supervision checklist show skills under observation which are likely to be CBAs’ best rather than their routine way of working, but this data provides evidence of the CBAs’ core competencies and highlights changes over time. The low supervision coverage in Pakistan meant we had fewer checklists than we had for India, and CBAs with more supervisory visits are over-represented in the data. There was good agreement around what typical home visits looked like both between data collection methods and between respondent groups which suggests our overall findings are valid. We were not able to accurately determine the content of the LHW home visits to identify the extent to which SPRING messages were delivered, but evidence from multiple sources suggest that fidelity was low. We utilized several qualitative methods and a variety of respondents enabling us to gain different points of views and experiences and to triangulate our findings. In the qualitative component transferability was enhanced by having multiple study sites, purposive sampling, sampling to saturation and reflexivity among those collecting and analyzing the data. Despite this the findings may not be transferrable to settings with very different contexts and there is likely to have been some social desirability bias (46, 47).

In conclusion, we identified several factors that could have improved implementation including: maximizing coverage and supervision by improving how visits and field supervision are scheduled and monitored through the use of feedback loops and course correction, using alternative delivery strategies such as group meetings when delivery by existing CBAs is unlikely to be feasible, identifying and focusing on core intervention components such as coaching during training and supervision, acknowledging that ECD skills are new for CBAs and take time and support to develop and a focus on barriers to family uptake such as integrating ECD into daily activities and highlighting the long term impact of the behaviors and how they are different from existing interaction and play. This can be achieved by building in on-going adaptation, improvement and responsive feedback into implementation to refine the intervention and maximize impacts rather than taking a linear approach to implementation.

## Data availability statement

The datasets presented in this article are not readily available, but data are available upon reasonable request. The qualitative data are confidential considering that participants could be identified if their interviews are read in full. Requests to access the datasets should be directed to z.hill@ucl.ac.uk.

## Ethics statement

This study, involving human participants, was reviewed and approved by London School of Hygiene & Tropical Medicine Research Ethics Committee (UK), Ethical Review Committees at the Human Development Research Foundation (Pakistan) and the Sangath Institutional Review board (India). Approval was also granted by the Indian Council of Medical Research’s Health Ministry Screening Committee (HMSC). The patients/participants provided their written informed consent to participate in this study.

## Author contributions

ZH, S Sikander, BA, RR, JS, AH, AR, BK and GD designed the SPRING trial and evaluation and/or inputted into the design of the process evaluation. S Sikander, S Soremekun, RR and SA assisted with the quantitative data collection and analysis. ZH, GD, and SZ oversaw the qualitative data collection and analysis. DK, NP, S Saleem, DV, and KS conducted the interviews and inputted into their interpretation. ZH wrote the first draft of the manuscript which was then reviewed by all authors. All authors contributed to the article and approved the submitted version.

## Conflict of interest

The authors declare that the research was conducted in the absence of any commercial or financial relationships that could be construed as a potential conflict of interest.

## Publisher’s note

All claims expressed in this article are solely those of the authors and do not necessarily represent those of their affiliated organizations, or those of the publisher, the editors and the reviewers. Any product that may be evaluated in this article, or claim that may be made by its manufacturer, is not guaranteed or endorsed by the publisher.
